# Autoantibodies against interleukin-1 receptor antagonist in Still’s disease in adult patients, data from a multicenter cohort study

**DOI:** 10.3389/fimmu.2026.1957327

**Published:** 2026-10-02

**Authors:** Piero Ruscitti, Elvira Favoino, Federica Viapiano, Giulia Sgarlata, Marta Vomero, Letizia Pia Di Corcia, Francesco Maria Mariani, Paola Cipriani, Roberto Giacomelli, Federico Perosa

**Affiliations:** 1 Department of Biotechnological and Applied Clinical Sciences, University of L’Aquila, L’Aquila, Italy; 2 Rheumatic and Systemic Autoimmune Diseases Unit, Department of Interdisciplinary Medicine, University of Bari Medical School, Bari, Italy; 3 Fondazione Policlinico Universitario, Rome, Italy; 4 Research and Clinical Unit of Immunorheumatology, Department of Medicine and Surgery, Università, Rome, Italy

**Keywords:** adult onset Still’s disease, anti-IL-1Ra autoantibodies, autoantibodies, autoinflammatory disease, Still’s disease

## Abstract

**Objectives:**

Emerging evidence supports the presence of autoantibodies targeting interleukin-1 receptor antagonist (IL-1Ra), a regulator of IL-1 signaling, in adult-onset Still’s disease (AOSD). However, existing data are limited by small cohorts and potential confounding due to prior exposure to IL-1 inhibitors. This study aimed to develop a simple and reproducible indirect ELISA for detecting anti-IL-1Ra antibodies and to assess their frequency and immunological features in IL-1 inhibitor-naïve AOSD patients.

**Methods:**

Serum samples from IL-1 inhibitor-naïve AOSD patients (n=46) and healthy controls (n=26) were analyzed using an indirect ELISA based on recombinant IL-1Ra (anakinra) as antigen. Specificity was confirmed by dot blot. Anti-IL-1Ra IgG were affinity-purified from a high-titer patient and characterized by ELISA and SDS-PAGE. Epitope mapping was performed using phage display peptide libraries, followed by sequence alignment and structural mapping onto native IL-1Ra.

**Results:**

Anti-IL-1Ra antibody levels were significantly higher in AOSD patients than in healthy controls (p=0.003), with a greater proportion showing high titers (p<0.001). Levels did not differ between active and inactive AOSD patients. Purified antibodies displayed specific dose-dependent binding to IL-1Ra. Phage display identified two peptide motifs corresponding to linear epitopes mapped to surface-exposed regions of the native protein, indicating recognition of unmodified IL-1Ra domains.

**Conclusions:**

Anti-IL-1Ra antibodies are detectable in IL-1 inhibitor-naïve AOSD patients using a standardized ELISA. The identification of distinct linear epitopes suggests a heterogeneous autoimmune response targeting IL-1Ra and may support a role for adaptive immunity within the Still’s disease spectrum. Further studies are needed to define their functional and clinical relevance.

## Introduction

Still’s disease is a rare systemic inflammatory disorder characterized by high daily spiking fever, an evanescent salmon-colored skin rash, arthritis, and frequent multi-organ involvement (1). It encompasses both systemic juvenile idiopathic arthritis (sJIA) and adult-onset Still’s disease (AOSD), the latter being regarded as the adult counterpart of the disease (2, 3). Increasing evidence supports the concept that sJIA and AOSD represent a continuum within a single disease spectrum, rather than distinct clinical entities, as proposed in the “Still’s disease continuum” paradigm (2, 3). This concept is substantiated by overlapping clinical manifestations, comparable complication profiles, and convergent gene-expression signatures observed across age groups (4, 5). AOSD is a highly complex and heterogeneous condition, with clinical presentation varying according to the extent and severity of systemic and organ-specific involvement (6, 7). The pathogenesis of the disease remains incompletely elucidated and is widely considered to arise from a complex interplay of genetic susceptibility, environmental triggers, and dysregulated immune responses (8). Genetic association studies have identified multiple susceptibility loci within the HLA class I and II regions, along with variants in genes implicated in autophagy and inflammasome regulation (9–16). Moreover, infectious agents have been proposed as potential triggers of disease onset or exacerbations, including viral pathogens such as Epstein–Barr virus, cytomegalovirus, parvovirus B19, and SARS-CoV-2, as well as various bacterial pathogens (17–21). Immunopathologically, AOSD is characterized by marked activation of the innate immune system and excessive production of pro-inflammatory cytokines, including interleukin (IL)-1β, IL-6, IL-18, tumor necrosis factor-α, and chemokines such as CX3CL1 (21–27). This dysregulated immune response represents a central driver of systemic inflammation and tissue damage and constitutes a key pathogenic hallmark of the disease (22–26). Among inflammatory mediators, IL-1β plays a pivotal role in both disease initiation and perpetuation. Its pathogenic relevance is strongly supported by the rapid and sustained efficacy of IL-1-targeted therapies, including the recombinant IL-1 receptor antagonist anakinra and the monoclonal anti–IL-1β antibody canakinumab (26–33). However, direct measurement of circulating IL-1β remains technically challenging and offers limited clinical reliability (26, 34).

The IL-1 receptor antagonist (IL-1Ra) is a critical endogenous regulator of IL-1 signaling, essential for maintaining inflammatory homeostasis (35). Recently, autoantibodies against IL-1Ra have been identified in patients with AOSD and sJIA, leading to reduced circulating levels and impaired bioactivity of IL-1Ra, with consequent amplification of the inflammatory response (34). These autoantibodies have been associated with a hyperphosphorylated IL-1Ra isoform, which may act as a neoepitope capable of breaking immune tolerance (34). Nevertheless, current evidence remains limited by small cohort sizes and by the inclusion of patients previously treated with anakinra, a factor that may confound the interpretation of these findings.

The present study aimed to develop a simple, reproducible, and potentially standardizable indirect ELISA for the detection of anti-IL-1Ra antibodies and to apply it to a cohort of anakinra-naïve AOSD patients, with the goal of assessing the presence and potential pathogenic relevance of these autoantibodies independently of pharmacological exposure.

## Materials and methods

### Patients and clinical data

Forty-six patients fulfilling the Yamaguchi classification criteria for AOSD were recruited from the Department of Biotechnology and Applied Sciences, University of L’Aquila (n=24), and from the Immuno-Rheumatology Unit of Campus Bio-Medico University of Rome (n=22). Thus, patients were recruited from two tertiary referral centers using identical inclusion criteria. Serum collection, processing, storage, and laboratory analyses were performed according to standardized procedures. We focused on adult patients; no pediatric patients were involved in this study. All patients were naïve to anakinra at the time of sera collection. Therefore, inclusion criteria were age ≥18 years, fulfilment of the widely used Yamaguchi criteria for AOSD, and absence of previous exposure to anakinra at the time of serum collection. Patients not fulfilling Yamaguchi criteria or previously treated with anakinra were excluded. Serum samples were obtained from existing biorepositories of the participating centers and were analyzed retrospectively for the purposes of the present study. As healthy controls (HCs), 26 healthy blood donors were included and compared with patients with AOSD. Selected HCs serum samples were collected before the onset of COVID-19 pandemic, in order to exclude potential confounding factors which could have been generated by SARS-CoV-2 infection or vaccination.

The local Ethics Committee (*Comitato Etico Azienda Sanitaria Locale 1 Avezzano/Sulmona/L’Aquila, L’Aquila, Italy*) approved the study (protocol number 0122353/17). It was conducted in accordance with Good Clinical Practice and the Declaration of Helsinki.

### Sera samples and reagents

Sera samples from the 46 patients with AOSD and from 26 HCs were stored at −80 °C until use. Bovine serum albumin (BSA) was obtained from MilliporeSigma (Burlington, MA, USA). Horseradish peroxidase (HRP)-conjugated goat anti-human IgG secondary antibodies were purchased from Jackson ImmunoResearch Laboratories (Avondale, PA, USA). Anakinra, a recombinant non-glycosylated human IL-1 receptor antagonist produced in Escherichia coli, was purchased from Swedish Orphan Biovitrum (SOBI), Stockholm, Sweden. Polyclonal human IgG preparations for intravenous use (IVIG, Intratec^®^) were purchased from Biotest (Dreieich, Germany). The c7c and 12-mer PDPLs were purchased from New England Bio Lab (Beverly, MA).

### Anakinra-based ELISA for the detection of anti-IL-1Ra antibodies

Ninety-six–well polystyrene microtiter plates were coated overnight at 4 °C with anakinra at the concentration of 80 µg/mL. Wells coated with the same amount of BSA were used as negative controls. Plates were washed with phosphate-buffered saline containing 0.05% Tween-20 (PBS-T20) and blocked with PBS containing 0.5% BSA for 1 h at room temperature. Serum samples were diluted 1:50 in PBS-BSA and added in duplicate (50 µL per well), followed by incubation for 3 h at room temperature. After four washes with PBS-T20, HRP-conjugated goat anti-human IgG secondary antibodies were added and incubated for 1 h at room temperature. Plates were washed, and color development was achieved using o-phenylenediaminedihydrochloride in citrate buffer (pH 5) with hydrogen peroxide. The reaction was stopped with 2 N sulfuric acid, and absorbance was measured at 490 nm. Specific binding was calculated by subtracting absorbance values obtained from BSA-coated wells from those obtained from anakinra-coated wells. Antibody levels were expressed as percentage binding relative to a reference positive control serum (pt1), defined as 100% binding.

### Dot blot analysis

Anakinra and BSA were spotted onto nitrocellulose membranes at concentrations of 5 µg, 2.5 µg, and 1.25 µg per dot. Membranes were blocked with 5% dry-milk in PBS-T20 and incubated with patient sera, diluted 200 times in 5% dry-milk in PBS-T20, for 3 h at room temperature under gentle agitation. After washing, membranes were incubated with HRP-conjugated goat anti-human IgG secondary antibodies. Immunoreactive dots were detected using Clarity™ Western ECL Substrate (Bio-Rad) and imaged with a ChemiDoc imaging system.

Because no validated cut-off values for anti-IL-1Ra antibody levels are currently available, antibody titers were additionally categorized according to their distribution within the study population. The highest quartile (4th quartile) was used to identify subjects with relatively elevated antibody levels. In addition, formal plate randomization and blinded sample analysis were not performed since beyond the primary scope of the present study.

### Affinity purification of anti-IL-1Ra antibody

Anakinra was conjugated to AffiGel 15 (Bio-Rad Laboratories, Hercules, CA, USA) at a concentration of 1 mg/ml of resin, following the manufacturer’s instructions, and used to purify human anti-anakinra IgG as previously described (36). Briefly, 10 ml of serum from pt1, selected for high binding avidity to anakinra as determined by ELISA, were diluted 1:1 in PBS, repeatedly passed through a BSA-conjugated AffiGel 15 column, and subsequently applied to an anakinra-conjugated column. Bound IgG were eluted, dialyzed overnight against PBS, and concentrated by lyophilization. Antibody concentration was determined by UV absorption. The purity of the purified antibodies was assessed by SDS–PAGE, as previously described (37).

### Panning of a phage display peptide libraries and nucleotide sequence analyses

To define the amino acid motif recognized by purified anti-anakinra Ab, panning of phage display libraries expressing 7-mer cyclic and 12-mer linear peptides with anti-anakinra Ab was performed as previously described (38). For each panning round, phage particles (2 × 10^11) were sequentially incubated with IVIG-coated protein A– and G–Sepharose to remove phages recognizing IgG isotypic or allotypic determinants. Unbound phages were then incubated with Sepharose beads coated with affinity-purified anti-IL-1Ra antibodies. After washing, bound phages were eluted with 0.2 M glycine (pH 2.8), and the eluate was immediately neutralized with 1 M Tris-HCl (pH 9.1). DNA from selected phage clones was sequenced by Eurofins Genomics (Ebersberg, Germany). Nucleotide sequences encoding the peptide inserts were translated using the ExPASy DNA Translate tool (http://web.expasy.org/translate/), and the resulting amino acid sequences were obtained. Antigenic motifs were identified by aligning the peptide sequences with the Multalin Protein tool available at the Pole Bio-InformatiqueLyonnaise (http://npsa-pbil.ibcp.fr/cgi-bin/npsa_automat.pl?page=/NPSA/npsa_multalin.html).

Sequence similarities were assessed according to standard amino acid conservation criteria (identical, conserved, and semi-conserved substitutions). Because the reactive phage clones clustered into only two unique peptide sequences, consensus similarity motifs were derived by aligning each peptide sequence with the human IL-1Ra amino acid sequence and identifying the residues showing sequence conservation or similarity.

### Phage-based competition assay

The competition assay was performed by mixing 50 μL of twofold serial dilutions of Pc.1 or Pc.2 phage clone supernatant, starting from undiluted supernatant, with an equal volume of purified anti-IL-1Ra antibodies (2.5 μg/ml). After incubation for 1 h at 25 °C, the mixtures were added to plates previously coated with anakinra. After incubation for 1 h at 25 °C, the mixtures were transferred to anakinra-coated plates and incubated for 3 h at 25 °C. Antibody binding was then detected as described above. The rituximab-specific phage clone Rp15-C was used as a negative control (39). Percentage inhibition was calculated as follows: % inhibition = [(OD_490_ without inhibitor − OD_490_ with inhibitor)/OD_490_ without inhibitor] × 100.

### Structural visualization and epitope mapping

Structural visualization and mapping were performed using the MOL* web-based molecular graphics viewer (MOLSTAR). The three-dimensional structure of human native mature IL-1Ra was obtained from the Protein Data Bank (PDB ID: 1IRP) and rendered as a solvent-accessible surface. The IL-1Ra sequence segments corresponding to residues 124–130 and 134–140 of the mature form (residues 149–155 and 159–165 of the precursor form), identified by sequence alignment as similar to Pc.1 and Pc.2 peptides, respectively, were selected and highlighted on the protein surface. Figures were exported directly from MOL*.

### Statistical analysis

Statistical analyses were performed using SPSS software version 21 (IBM, USA). Normality was assessed and data were analyzed using the non-parametric Mann–Whitney U test or Kruskal-Wallis’s test. Data are presented as medians. Because no validated cut-off values for anti-IL-1Ra antibody levels are currently available, a quartile-based exploratory analysis was performed. The threshold corresponding to the 75th percentile (upper quartile) was used to identify subjects with relatively elevated anti-IL-1Ra antibody levels. This threshold was used for descriptive purposes only and was not intended to define a clinically validated category of positivity. Differences in proportions were analyzed using Chi-square test. Given the exploratory nature of the study and the limited number of predefined comparisons, adjustment for multiple testing was not performed. No missing data were present in the analyzed dataset. A p value<0.05 was considered statistically significant.

## Results

### Anti-IL-1Ra antibody levels in AOSD and relationship with disease activity

In this study, 46 patients affected by AOSD who had not been previously treated with anakinra were assessed. The clinical characteristics of the cohort are described in **Table 1**. Patients were divided in “active” or “inactive”. The “active” patient was codified by the presence of disease signs and symptoms or the need of any additional therapy (or any increased drug dosages) to control the clinical picture. The “inactive” patient was defined as the absence of disease signs and symptoms, considered by the physician in charge of the patients to be linked with disease activity, together with the normalization of laboratory inflammatory markers for at least two consecutive months, regardless of therapy. At the time of study design, no universally accepted definition of active versus inactive AOSD was available. Therefore, disease activity status was defined according to predefined clinical and laboratory criteria routinely used in clinical practice. In addition, disease activity was assessed by the treating physician after exclusion of alternative causes of systemic inflammation, including active infections and other clinically evident inflammatory conditions. Detailed clinical and laboratory characteristics of both groups are reported in **Table 1**.

**Table 1:** Descriptive characteristics of assessed patients with AOSD grouped in active and inactive.

Clinical characteristics	34 active patients	12 inactive patients
Age, years, mean±sd	38.4 ± 14.6	39.9 ± 15.6
Male sex, n (%)	16 (47.1)	6 (50.0)
Fever, n (%)	34 (100)	0 (0.0)
Arthritis, n (%)	28 (82.3)	0 (0.0)
Skin Rash, n (%)	26 (76.4)	0 (0.0)
Myalgia, n (%)	20 (58.8)	0 (0.0)
Hepatomegaly, n (%)	18 (52.9)	8 (66.7)
Sore throat, n (%)	18 (52.9)	0 (0.0)
Lymphadenomegaly, n (%)	17 (50.0)	2 (16.7)
Splenomegaly, n (%)	17 (50.0)	4 (32.3)
Pericarditis, n (%)	10 (29.4)	0 (0%)
Pleuritis, n (%)	9 (26.5)	0 (0%)
CRP, mg/L, median (IQR)	56.0 (76.5)	0.9 (0.8)
ESR, mm/h, median (IQR)	87.0 (54.0)	5.0 (6.0)
Ferritin, ng/mL, median (IQR)	1343.0 (867.0)	264.0 (256.0)
Low dose of GCs, n (%)	6 (17.6)	5 (41.7)
High dose of GCs, n (%)	28 (82.4)	0 (0.0)
Methotrexate, n (%)	16 (47.1)	8 (66.7)
IL-6 inhibitor, n (%)	0 (0.0)	7 (58.3)

AOSD, adult onset Still’s disease; n, number of patients; SD, standard deviation; IQR, interquartile range; CRP, C reactive protein; ESR, erythrocyte sedimentation rate; GCs, glucocorticoids.

Sera levels of anti-IL-1Ra antibodies were assessed in included patients and in 26 HCs using an indirect ELISA assay with anakinra used as the antigen. Representative ELISA results are shown in **Figure 1A**. Explanatory serum samples were selected to illustrate high, intermediate, and low/negative anti-IL-1Ra reactivity among AOSD patients and the absence or minimal reactivity observed in HCs. Samples were chosen for illustrative purposes only and were not selected according to specific clinical characteristics. Assay specificity was subsequently further evaluated and confirmed by immunodot analysis (**Figure 1B**). Sera identified as positive by ELISA showed a clear, specific, and dose-dependent reactivity toward anakinra in this assay, while no binding was observed against BSA, which was included as a negative control. In contrast, sera that tested negative by ELISA did not exhibit any detectable signal in the immunodot analysis, either against anakinra or BSA. Overall, these results support the reliability of the ELISA assay in distinguishing between positive and negative samples and confirm the specificity of the antibody response detected. Sera from patient P1, which exhibited high binding activity, was used as the reference standard for expressing binding reactivity as a percentage. As shown in **Figure 2A**, sera levels of anti-IL-1Ra antibodies were significantly higher in patients with AOSD compared with HCs (p=0.003). Using the predefined upper-quartile threshold (75th percentile, corresponding to 24.7% binding), 16/46 (34.8%) AOSD patients were classified as having relatively elevated anti-IL-1Ra antibody levels. Antibody levels within this upper-quartile subgroup ranged from 24.7% to 82.47% binding (median 32.83% binding) and were significantly more frequent than in healthy controls (p<0.001) (**Figure 2B**). Thus, a significantly larger proportion of AOSD patients fell within the highest quartile of anti-IL-1Ra antibody levels compared with HCs. Within the AOSD cohort, no significant differences in anti-IL-1Ra antibody levels were observed between patients with active disease and those with inactive disease (p=0.784) (**Figure 2C**).

**Figure 1:**
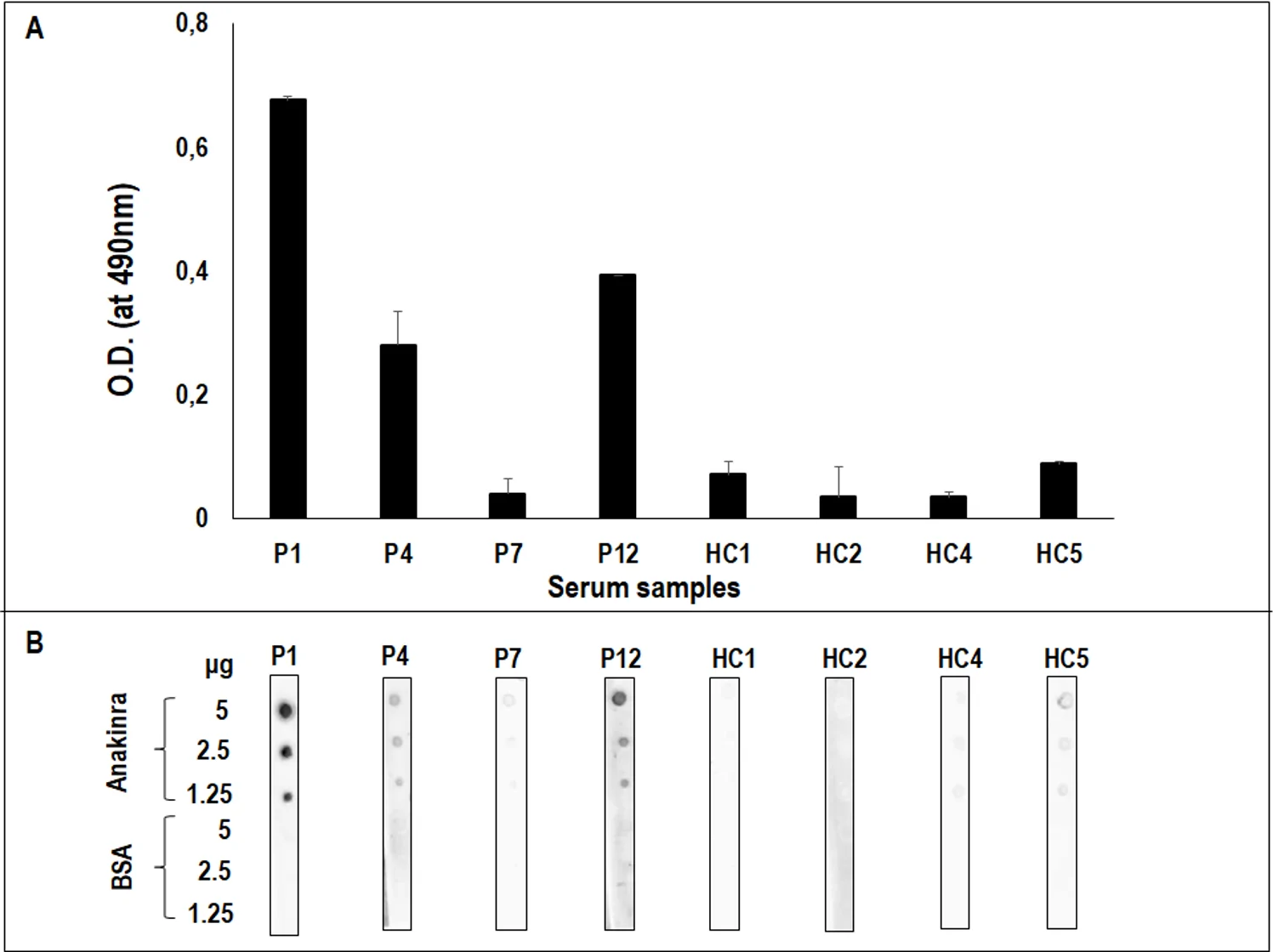
Detection of anti–IL-1Ra antibodies. **(A)** Serum samples from patients with adult-onset Still’s disease (AOSD) and healthy controls (HCs) were added to wells previously coated with anakinra or with bovine serum albumin (BSA) used as a negative control. After incubation, antibody binding was detected using HRP-conjugated goat anti-human IgG, and results were expressed as optical density (OD) at 490 nm. Specific binding was calculated by subtracting absorbance values obtained from BSA-coated wells from those obtained from anakinra-coated wells. Explanatory serum samples were selected to illustrate high, intermediate, and low/negative anti-IL-1Ra reactivity among AOSD patients and the absence or minimal reactivity observed in healthy controls. Samples were chosen for illustrative purposes only and were not selected according to specific clinical characteristics. Data are presented as the mean ± standard deviation of two independent ELISA experiments. **(B)** Anakinra and BSA were spotted onto nitrocellulose membranes at decreasing amounts (5, 2.5, and 1.25 µg) and incubated with serum samples. Bound antibodies were detected using HRP-conjugated goat anti-human IgG. Representative results are shown.

**Figure 2:**
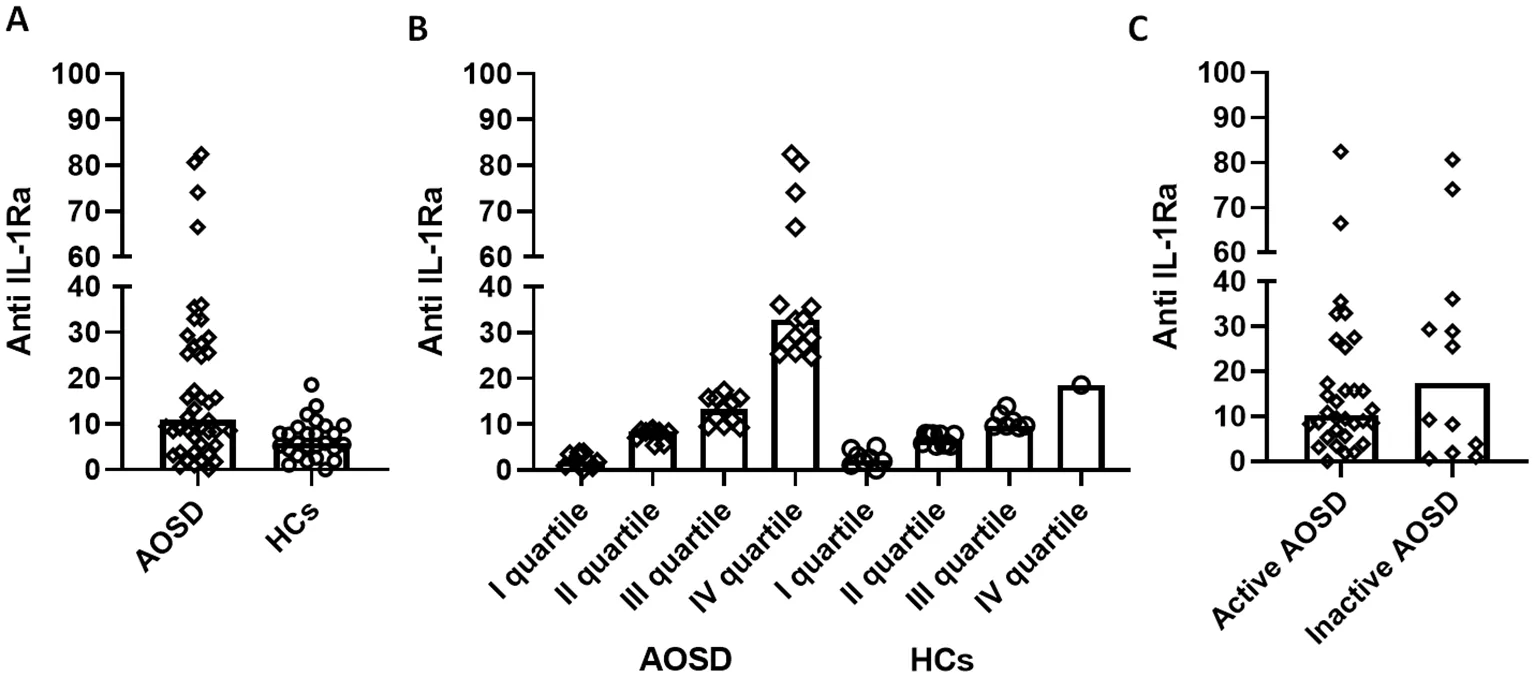
Anti-IL-1Ra antibody levels are elevated in adult-onset Still’s disease (AOSD) independently of disease activity. **(A)** Sera levels of anti-IL-1Ra antibodies were measured by indirect ELISA in patients with AOSD and healthy controls (HCs). Antibody levels were expressed as percentage binding relative to a reference positive control serum. Sera levels of anti-IL-1Ra antibodies were significantly higher in patients with AOSD compared with HCs (p=0.003). **(B)** A significantly larger proportion of AOSD patients (34.8%) exceeded the predefined upper-quartile threshold for anti-IL-1Ra antibody levels (p<0.001). **(C)** Within the AOSD cohort, no significant differences in anti-IL-1Ra antibody levels were observed between patients with active disease and those with inactive disease (p=0.784).

### Purification and characterization of anti-IL-1Ra antibodies

Anti-IL-1Ra antibodies were purified from pooled serum samples with high reactivity from AOSD patient P1 collected over a one-year period. Ig purification was performed by affinity-chromatography on anakinra-coupled Affigel column. Coomassie blue–stained SDS–PAGE showed the purity and IgG nature of the purified anti–IL-1Ra antibodies (**Figure 3A**). The specificity of anti-IL-1Ra IgG for anakinra was examined by ELISA. As shown in **Figure 3B**, purified anti-IL-1Ra antibodies reacted with anakinra in a dose-dependent manner. The specificity of this reactivity was demonstrated by the lack of binding of purified anti–IL-1Ra antibodies to BSA and of IVIG to both anakinra and BSA (**Figure 3B**).

**Figure 3:**
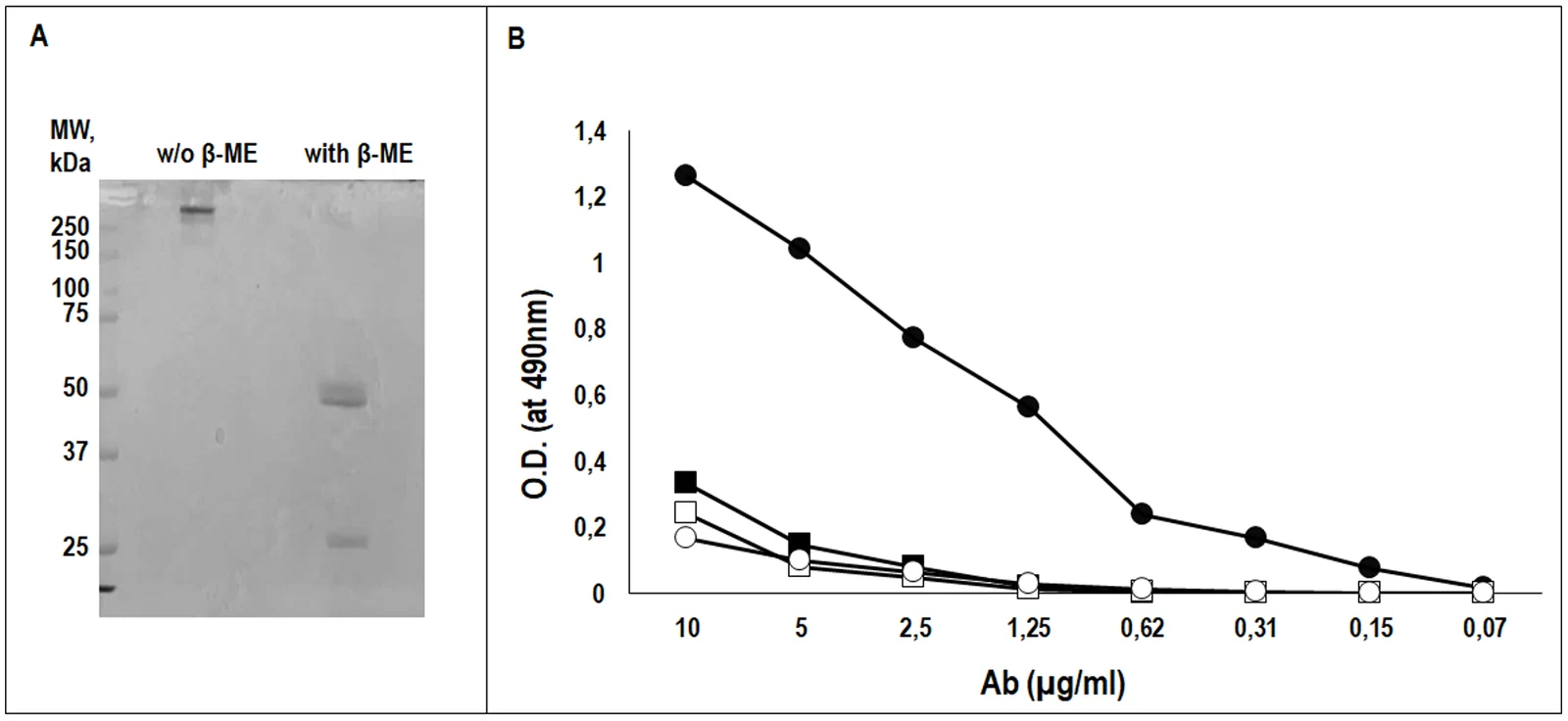
Purification and specificity of anti–IL-1Ra antibodies. **(A)** Anti–IL-1Ra antibodies were purified from serum of AOSD patient P1 by affinity chromatography using anakinra-conjugated Affi-Gel columns. Purified antibodies (5 μg/lane) were run onto SDS–PAGE under non-reducing and reducing conditions and stained with Coomassie brilliant blue. **(B)** The reactivity of purified anti–IL-1Ra antibodies with anakinra was assessed by indirect ELISA assay, incubating different concentrations of purified antibodies with anakinra (closed circle •) or bovine serum albumin (BSA) (open circle ○). Human immunoglobulins for intravenous use (IVIG) were used as negative controls and assessed for binding to anakinra (closed square ◼) and bovine serum albumin (BSA) (open square □). Bound IgG was detected using HRP-conjugated goat anti-human IgG and o-phenylenediamine. Data are representative of duplicate wells.

### Characterization of anti-IL-1Ra fine specificity by PDPL

Purified anti-IL-1Ra IgG were used to screen a mixed linear 12-mer and cyclic 7-mer PDPL for the selection of antigen-specific phage clones. In each round of panning, IVIG were included to remove phage clones binding to isotypic or allotypic determinants. ELISA screening of 44 randomly selected phage clone supernatants identified 7 (15.9%) anti-IL-1Ra-specific phage clones. Phage clones insert sequence analysis led to the identification of two distinct phage clusters, each characterized by identical peptide sequences (**Table 2**). Thus, all reactive clones converged into two unique peptide sequences, designated Pc.1 and Pc.2, suggesting preferential enrichment of a limited number of antibody-recognized mimotopes. Phage cluster 1 (Pc.1), accounting for 28.5% of reactive clones, expressed the cyclic peptide acSMLYDKSc and showed weak but specific reactivity with purified anti-IL-1Ra IgG, while no binding was observed with IVIG. The second cluster (Pc.2), representing the majority of reactive clones (71.5%), expressed the cyclic peptide acTNRAHQWc and exhibited strong reactivity with anti-IL-1Ra antibodies, with no detectable reactivity to IVIG. These findings indicate preferential recognition of the Pc.2 peptide by anti-IL-1Ra antibodies.

**Table 2:** Reactivity and deduced amino acid sequences of anti-IL-1Ra Ab-specific phage clones.

Phage clone insert #	Phage cluster #	Phage denomination	Reactivity (O.D.490^nm^)^a)^		Deduced amino acid sequence ^)^
			Anti-IL1Ra IgG	IVIG	
1	1	P.3	0.389±0.005	0.011±0.000	acSMLYDKSc
2	1	P.7	0.397±0.002	0.011±0.001	acSMLYDKSc
2	2	P.21	1.122±0.014	0.043±0.011	acTNRAHQWc
3	2	P.26	1.978±0.023	0.022±0.002	acTNRAHQWc
4	2	P.28	1.234±0.006	0.031±0.012	acTNRAHQWc
5	2	P.32	1.342±0.014	0.013±0.003	acTNRAHQWc
6	2	P.43	1.211±0.008	0.023±0.002	acTNRAHQWc

^a^Mean ± standard deviation.

To verify the specificity of the selected mimotopes, competition experiments were performed using Pc.1 and Pc.2 phage clone supernatants. As shown in **Figure 4**, undiluted Pc.2 and Pc.1 supernatants inhibited purified anti-IL-1Ra antibody binding to anakinra by approximately 18% and 7%, respectively. Inhibition decreased with increasing phage supernatant dilution and was no longer detectable at the fourfold dilution. In contrast, the Rituximab-specific phage clone Rp15-C did not show any inhibitory activity. These results support the specific recognition of Pc.1 and Pc.2 by a subset of the purified anti-IL-1Ra antibodies, with Pc.2 displaying greater inhibitory activity than Pc.1.

**Figure 4:**
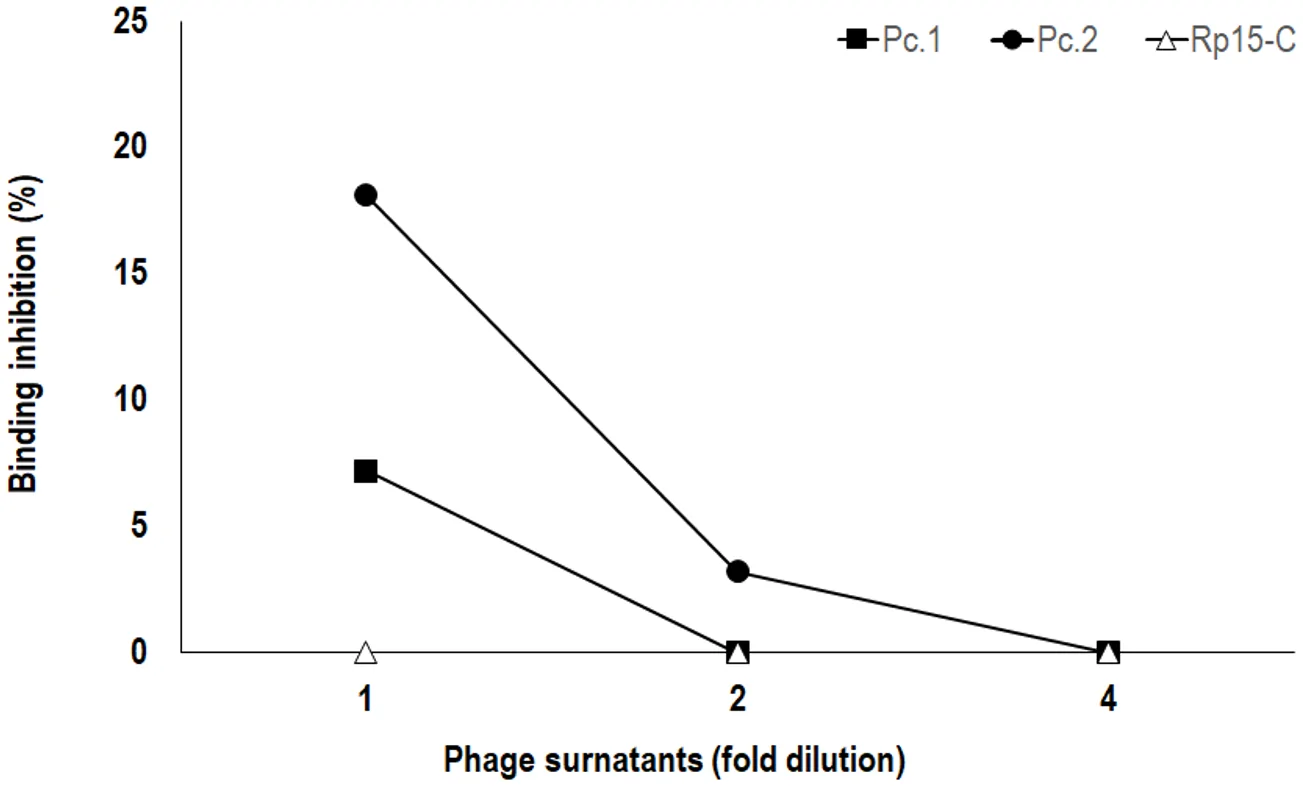
Inhibition of anti-IL-1Ra antibody binding to anakinra by phage clone peptide supernatants. Purified anti-IL-1Ra antibodies were preincubated with undiluted or twofold serially diluted Pc.1 or Pc.2 phage clone supernatants before being added to anakinra-coated plates. The Rituximab-specific phage clone Rp15-C was used as a negative control. Results are expressed as percentage inhibition relative to antibody binding in the absence of inhibitor.

To identify candidate IL-1Ra regions recognized by the selected mimotopes, the Pc.1 and Pc.2 peptide sequences were aligned with the human IL-1Ra sequence. Based on identical, conserved and semi-conserved amino acid residues, Pc.1 displayed partial similarity with mature IL-1Ra residues 124–130, yielding the consensus similarity motif AMxxDQx, whereas Pc.2 displayed sequence similarity with mature IL-1Ra residues 134–140 (TNMPDEG), yielding the consensus similarity motif TNxxxEx (**Table 3**).Mapping of the Pc-derived peptide-related sequences onto the three-dimensional structure of the native mature IL-1Ra showed that the regions corresponding to residues 124–130 and 134–140 are exposed on the protein surface (**Figure 5**), consistent with their potential accessibility to antibody recognition.

**Table 3:** Sequence similarity between Pc-derived peptides and IL-1Ra fragments.

Denomination	Amino acid sequence
Pc.1	SMLYDKS
Mature IL-1Ra^124-130^	AMEADQP
Similarity^a^	:* *:.
*Motif*	AMxxDQx
Pc.2	TNRAHQW
Mature IL-1Ra^134-140^	TNMPDEG
Similarity	** ..:
*Motif*	TNxxxEx

^a^ Similarity symbols indicate the degree of residue conservation (*, identical residues; : conserved substitutions; ., semi-conserved substitutions).

**Figure 5:**
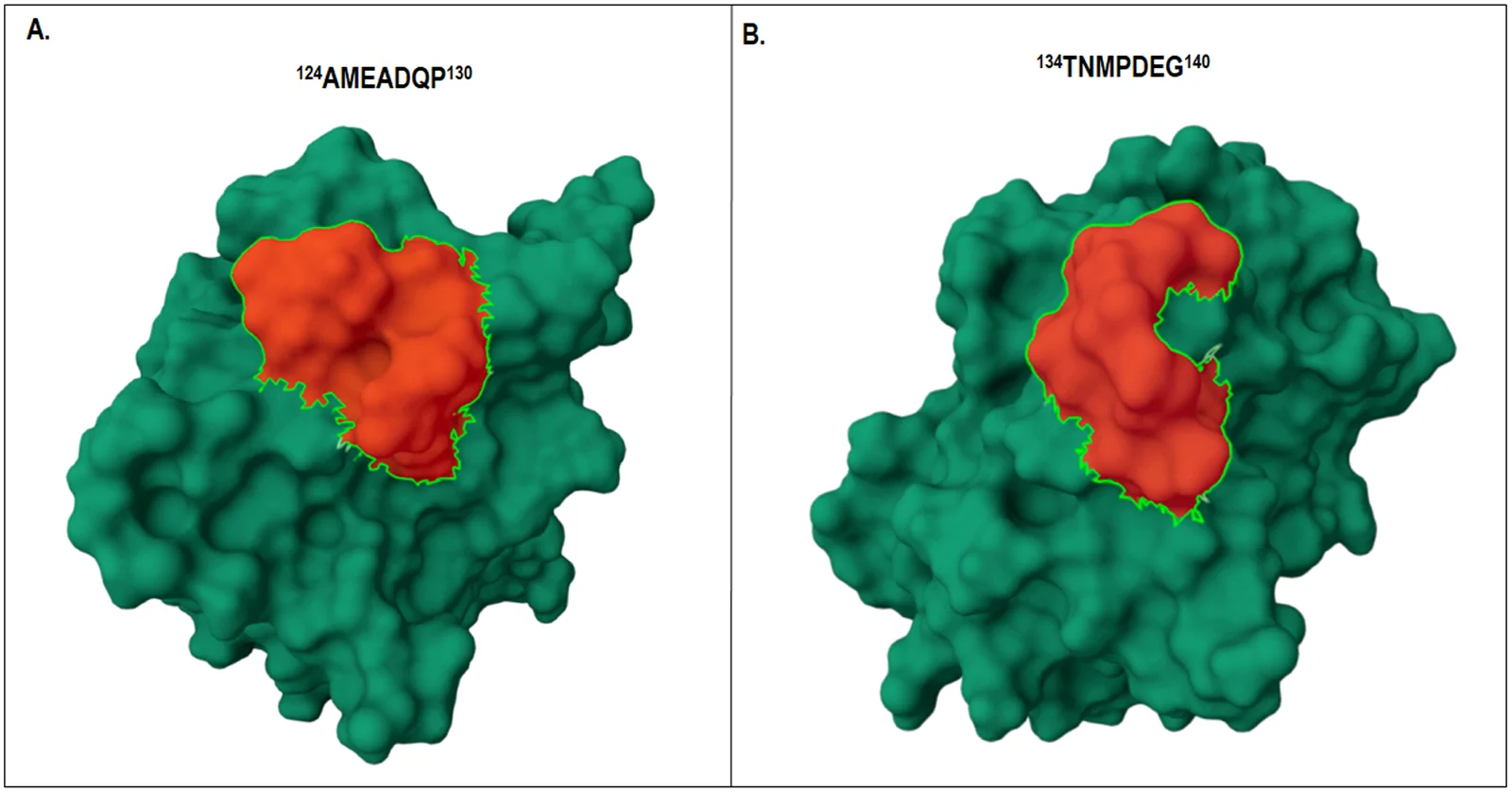
Surface-exposed epitopes of IL-1Ra recognized by autoantibodies. The three-dimensional structure of human IL-1Ra was visualized using the MOL* molecular graphics viewer. Regions spanning residues 124-130 **(A)** and 134-140 **(B)** of mature IL-1Ra, identified by alignment with phage display–derived peptides, are highlighted on the protein surface.

## Discussion

In the present study, we developed and applied an indirect ELISA assay based on anakinra as antigen for the detection of circulating anti-IL-1Ra antibodies in patients with AOSD. Using this approach, we observed significantly higher sera levels of anti-IL-1Ra antibodies in patients with AOSD compared with HCs, supporting the presence of an immune response directed against the IL-1 regulatory axis. In almost one-third of patients a high titer of anti-IL-1Ra antibodies was observed. The analysis of a relatively large and well-characterized cohort represents a major strength of this study, particularly considering the rarity of AOSD and the limited size of cohorts reported in previous investigations. Importantly, all patients included were naïve to anakinra thereby minimizing the potential confounding effect of therapy-induced immunogenicity, which has been described for biologic agents used in immune-mediated inflammatory diseases, including IL-1-targeted therapies (40–42). However, the use of the highest quartile was intended as an exploratory, distribution-based approach and should not be interpreted as a validated threshold for antibody positivity or clinical significance.

Despite the clear difference observed between patients with AOSD and HCs, anti-IL-1Ra antibody levels did not significantly differ between patients with active disease and those in remission. This finding suggests that these antibodies may not directly reflect current inflammatory activity but rather represent a relatively stable immunological feature or a form of persistent immune memory. Such a pattern aligns with the clinical heterogeneity of AOSD and supports the concept that immune dysregulation may persist independently of overt disease activity. In this context, growing evidence indicates that the adaptive immune system also contributes to the pathogenesis of sJIA and AOSD (43–45). In particular, a skewing of naive T helper cell differentiation toward T follicular helper (TFH) and T peripheral helper (TPH) phenotypes has been described in patients with sJIA, leading to increased production of IL-21 (46). In combination with the concomitant expression of PD-1, ICOS, and CXCR5, IL-21 may further promote the polarization of naive T helper cells toward TFH/TPH phenotypes, thereby enhancing B cell activation, plasmablast differentiation, and IgG production (46). Consistently, disease-promoting B cell subsets, including naive B cells, double-negative B cells, and plasmablasts, have been reported to be expanded in the peripheral blood of patients with AOSD (47). Taken together, these findings, along with our observation of anti-IL-1Ra antibodies, may support the concept of a distinctive pathogenic scenario in AOSD. This landscape may be interpreted within an inflammatory continuum that involves both innate and adaptive immunity, positioning AOSD at the crossroads between autoinflammatory and autoimmune disorders (2, 43, 44, 48).

To further characterize the anti-IL-1Ra immune response, we investigated antibody fine specificity by purifying polyclonal IgG from a representative patient with AOSD using anakinra as affinity ligand and performing phage display-based epitope mapping. This strategy led to the identification of short linear peptide motifs corresponding to distinct regions of the IL-1Ra sequence. Sequence alignment and structural mapping demonstrated that these motifs localize to surface-exposed regions of the native mature IL-1Ra, consistent with their accessibility for antibody recognition. Although the identified regions are surface-exposed and therefore accessible to antibody recognition, the present study was not designed to determine whether they overlap with, or influence, IL-1 receptor-binding interfaces. Consequently, we cannot establish whether antibodies directed against these regions might interfere with the biological activity of IL-1Ra. Future structural and functional studies, including receptor-binding and signaling assays, will be required to clarify the potential biological significance of these antibody-recognition regions. Because anakinra is a recombinant IL-1Ra produced in Escherichia Coli and lacks post-translational modifications such as glycosylation or phosphorylation (49–51), the antibodies isolated in this study necessarily recognize unmodified regions of the IL-1Ra molecule. These findings indicate that anti-IL-1Ra inflammatory response in AOSD is not restricted to recognition of post-translationally modified protein isoforms but may also involve linear determinants present in the native mature protein. Nevertheless, because antibody detection and characterization were performed using recombinant anakinra as antigen, direct recognition of native human IL-1Ra was not formally demonstrated. Therefore, the identified regions should be interpreted as candidate native IL-1Ra-related antibody-recognition regions rather than definitive proof of physiological recognition of the endogenous protein.

Comparison with previous studies highlights the heterogeneity of the anti-IL-1Ra antibody response. Hoffmann et al. reported that anti-IL-1Ra antibodies in Still’s disease preferentially recognize linear IL-1Ra peptides spanning G^98–N^116 and F^125–G^143 (34). In that study, epitope mapping was performed using FLAG-tagged IL-1Ra fragments recombinantly expressed in HEK293 cells. Although the peptide sequences themselves were unmodified, antibody reactivity was strongly associated with the presence of hyperphosphorylated IL-1Ra *in vivo*, suggesting that post-translational modification might have influenced the accessibility or immunogenicity of specific regions of the molecule. In contrast, the epitopes identified in the present study map to more C-terminal regions of the native IL-1Ra (149–155 and 159-165), which correspond to surface-exposed residues 124–130 and 134–140 of the mature protein. Taking together these observations, anti-IL-1Ra inflammatory response may involve the recognition of multiple, non-overlapping linear determinants across the IL-1Ra molecule, whose immunogenicity may be differentially influenced by protein modification and antigen presentation. Within this framework, the differential reactivity observed between the two Pc-derived peptides identified in this study should be interpreted with caution. Although Pc.2 showed stronger recognition than Pc.1, both peptides were selected using polyclonal antibodies purified from the same patient, excluding inter-individual variability. Pc.2 may exhibit stronger antibody binding because it represents an epitope preferentially targeted by the polyclonal immune response. Alternatively, its cyclic structure, when presented in the phage display system, may adopt a conformation and exposure that favor more efficient antibody engagement compared with Pc.1. These features may influence peptide selection in the experimental setting and may not fully reflect binding behavior toward the native IL-1Ra protein. The mild inhibition observed in the competition assay may be explained by the polyclonal nature of the purified anti-IL-1Ra antibodies. Pc.1 and Pc.2 are likely recognized by only a subset of the antibody population, whereas other antibodies may target distinct determinants on anakinra that are not represented by the selected phage-displayed peptides. Consequently, individual phage clones would be expected to inhibit only a limited proportion of the overall polyclonal antibody binding to anakinra. At present, these mechanisms remain speculative and require further investigation. From a methodological perspective, the ELISA developed in this study differs from those used in previous reports investigating anti-IL-1Ra autoantibodies. Earlier studies primarily relied on IL-1Ra expressed in eukaryotic systems, often as tagged proteins immobilized on ELISA plates via tag-specific capture antibodies using total cell lysates (34, 52–54). While this approach allows detection of antibodies recognizing both native and post-translationally modified IL-1Ra isoforms, it may introduce antigen heterogeneity related to variable post-translational modification, expression levels, and antigen presentation. In contrast, the present assay is based on anakinra, a purified, non-tagged recombinant IL-1Ra lacking post-translational modifications, thereby reducing antigen heterogeneity and facilitating assay reproducibility and standardization. Importantly, this approach selectively identifies antibodies directed against unmodified regions of the IL-1Ra backbone and thus complements existing methodologies rather than replacing them.

Several limitations of this study should be acknowledged. First, functional experiments assessing the biological activity of anti-IL-1Ra antibodies, including their impact on circulating IL-1Ra levels or IL-1 signaling, were not performed. Furthermore, no functional studies were conducted to determine whether antibodies recognizing the identified IL-1Ra regions interfere with IL-1 receptor binding or modulate the biological activity of IL-1Ra. Therefore, the functional relevance of the identified antibody-recognition regions remains uncertain. In addition, formal sample-size or statistical power calculation was performed because the study was conceived as an exploratory investigation in a rare disease population and included all available eligible patients from the participating centers. Therefore, the study may have been underpowered to detect more subtle clinical associations. Furthermore, the ELISA developed in this study was based on recombinant E. coli-derived anakinra, which lacks post-translational modifications such as glycosylation and phosphorylation. Therefore, the assay selectively identifies antibodies directed against unmodified IL-1Ra and does not capture immune responses against modified IL-1Ra isoforms that have been implicated in previous studies (34, 52–54). Additionally, the study did not include validation experiments using independently produced native human IL-1Ra. Consequently, direct recognition of endogenous IL-1Ra by the detected antibodies remains to be formally demonstrated. Furthermore, antibody reactivity was expressed relative to a single highly reactive reference serum (P1), which was arbitrarily assigned a value of 100% binding. Although this approach facilitated normalization of the assay, the absence of an independent calibration standard may limit the comparability of antibody levels across different studies and assay platforms. Consequently, the pathogenic relevance of the detected antibodies and their relationship with antibodies recognizing post-translationally modified IL-1Ra remain uncertain. In addition, no orthogonal validation strategy or external cohort validation was available. Given the known methodological variability in anti-IL-1Ra antibody detection across different laboratories and assay platforms (55), the reproducibility and generalizability of our findings require further confirmation. Furthermore, the quartile-based classification adopted in the present study was used as an exploratory distribution-based approach in the absence of validated biological or clinical cut-off values for anti-IL-1Ra antibodies. Therefore, the identified threshold should not be interpreted as a clinically meaningful definition of antibody positivity or high titer. Although the assay was developed as a simple research tool for the detection of anti-IL-1Ra antibodies, a comprehensive analytical validation according to diagnostic assay development standards was beyond the scope of the present study. Consequently, parameters such as inter-assay variability, analytical range, formal assay acceptance criteria, and blinded reproducibility assessments were not systematically evaluated and should be addressed in future validation studies. In addition, epitope mapping was performed using affinity-purified polyclonal antibodies from a single representative patient and therefore may not reflect the full spectrum of antibody specificities present across the broader AOSD population. Furthermore, the identified antibody-recognition regions were inferred through phage-display peptide selection, sequence similarity analysis, and structural surface mapping rather than direct experimental determination of antibody-antigen interfaces. Consequently, these regions should be regarded as candidate antibody-recognition sites rather than definitively established epitopes. In addition, owing to the cross-sectional design, we were unable to investigate temporal changes in antibody levels, their relationship with disease evolution, therapeutic response, or long-term clinical outcomes. Additional longitudinal and multicenter studies combining functional assays and independent validation cohorts will be required to clarify the biological and clinical significance of anti-IL-1Ra antibodies in Still’s disease. Finally, patients were receiving different therapeutic regimens at the time of blood sampling, including glucocorticoids, methotrexate, and biologic agents. The potential influence of these treatments on anti-IL-1Ra antibody levels could not be formally assessed and therefore cannot be excluded.

In conclusion, this study may suggest the presence of circulating anti-IL-1Ra antibodies in AOSD using a simple and potentially standardizable assay and may propose that these antibodies may recognize unmodified regions corresponding to the native mature IL-1Ra sequence. In addition, future studies including larger numbers of antibody-positive patients will be required to determine the prevalence and reproducibility of these recognition regions across the broader AOSD population. The identification of distinct IL-1Ra-derived linear motifs underscores the complexity of tolerance breakdown in autoinflammatory diseases and may support a possible convergent pathogenic role for IL-1Ra-directed inflammatory response across Still’s disease continuum. Further longitudinal and functional studies will be required to clarify the clinical relevance, pathogenic potential, and therapeutic implications of anti-IL-1Ra autoantibodies.

## Data Availability

The raw data supporting the conclusions of this article will be made available by the authors, without undue reservation.
